# Decoupled Mechanical and Surface Deterioration Trajectories of Cement Mortars with Different Fine Aggregates Under Freeze–Thaw Exposure

**DOI:** 10.3390/ma19163480

**Published:** 2026-08-18

**Authors:** Feng Ji, Yuexiang Xing, Hengxuan Qiao, Jiaerheng Adelieti, Ziwei Yan, Gang Wang

**Affiliations:** 1 School of Mathematics and Physics, Xinjiang Institute of Engineering, Urumqi 830023, China; jf11@xjie.edu.cn (F.J.); 18195881013@163.com (Z.Y.); 2 Key Laboratory of Xinjiang Coal Resources Green Mining, Ministry of Education, Xinjiang Institute of Engineering, Urumqi 830023, China; 3 Xinjiang Zhongxin Construction Engineering Quality Testing Co., Ltd., Artux City 845350, China; 4 Emergency Management Science Research Institute of Xinjiang Uygur Autonomous Region, Urumqi 830011, China; 5 School of Energy Engineering, Xinjiang Institute of Engineering, Urumqi 830023, China

**Keywords:** cement mortar, alternative fine aggregate, freeze–thaw exposure, damage trajectory, compressive strength, mass loss, scanning electron microscopy

## Abstract

Fine-aggregate source can alter both the load-bearing response and surface scaling of mortar under freezing and thawing, but these responses are often reduced to a single durability ranking. This study compared coal gangue sand mortar (CGM), river sand mortar (RSM), desert sand mortar (DSM), and standard sand mortar (StSM) after 0, 25, and 50 freeze–thaw cycles (FTCs). Compressive strength and mass loss were measured using three replicate specimens per quantitative condition, while post-compression fragments were examined by scanning electron microscopy (SEM). The primary integrative analysis was a parameter-free two-dimensional damage-trajectory map that retained absolute compressive strength and mass loss as separate measured axes; a weighted coupled index was retained only as an auxiliary sensitivity check. Before cycling, the compressive strengths of StSM, RSM, DSM, and CGM were 68.13±0.48, 53.90±0.39, 19.47±0.33, and 6.49±0.07 MPa, respectively. After 50 FTCs, StSM retained the highest absolute strength (33.61±0.39 MPa) and the lowest mass loss (0.21±0.02%), whereas DSM retained 9.91±0.19 MPa and exhibited the highest mass loss (17.46±0.05%). The StSM trajectory moved primarily toward lower strength with negligible surface-material loss, while DSM moved toward both low residual strength and severe scaling. RSM showed substantial strength reduction followed by later-stage surface loss. CGM followed an atypical trajectory in which measured strength increased to 11.13±0.13 MPa while mass loss reached 9.67±0.04%; because age-matched non-frozen controls were unavailable, this apparent gain cannot be separated from continued hydration and specimen-age effects. The SEM images suggested pores, interfacial discontinuities, cracking, and matrix loosening, although some defects may have been induced or widened by compression. The trajectory representation exposed distinct deterioration modes without arbitrary weighting, whereas the calculated ordering of CGM and RSM in the auxiliary index changed with weighting and normalization choices. The results support reporting absolute residual strength and surface loss jointly when screening alternative fine aggregates for cold-region mortar.

## 1. Introduction

Cement mortar is one of the most widely used cement-based materials in masonry, plastering, repair and infrastructure engineering. As a major component of mortar, fine aggregate directly affects particle packing, water demand, pore structure, interfacial transition zone (ITZ) characteristics and, consequently, the mechanical properties and durability of hardened mortar. Natural river sand has traditionally been used as the preferred fine aggregate because of its relatively stable grading, rounded particle morphology and good compatibility with cement paste. However, the rapid growth of infrastructure construction has greatly increased the consumption of natural sand, leading to riverbed degradation, ecological disturbance and concerns over aggregate resource depletion [1,2]. Therefore, the development of alternative fine aggregates has become an important strategy for sustainable construction materials.

The performance of cement-based materials prepared with alternative aggregates is strongly governed by the physical properties of the aggregates. Poon et al. [3] demonstrated that the moisture condition of natural and recycled aggregates significantly affects the slump and compressive strength of concrete, indicating that aggregate water absorption and initial water state are critical factors when non-conventional aggregates are used. In another study, Poon et al. [4] found that the ITZ microstructure plays a decisive role in the compressive strength of recycled aggregate concrete; a loose and porous ITZ generally results in inferior mechanical performance, whereas a denser ITZ improves stress transfer between aggregate and paste. Kou and Poon [5] further reported that the durability of recycled aggregate concrete can be improved when the aggregate quality and pore structure are properly controlled. Long-term investigations also showed that recycled aggregate concrete can achieve acceptable mechanical and durability performance through appropriate mix design and the incorporation of supplementary cementitious materials [6,7]. These findings suggest that the evaluation of alternative fine aggregates should include not only strength development but also durability degradation and microstructural evolution.

Among the potential alternative fine aggregates, desert sand has received considerable attention because of its abundant reserves in arid and semi-arid regions. Al-Harthy et al. [8] investigated concrete made with fine dune sand and reported that the incorporation of dune sand affects workability, strength and water absorption. Luo et al. [9] showed that very fine dune sand particles influence the workability and strength of concrete through both filler effects and increased water demand. Zhang et al. [10] experimentally evaluated mortar and concrete containing very fine desert sand and reported that desert sand can be used as a fine aggregate for general civil engineering applications. Yan et al. [11] optimized the mix proportion of desert sand concrete and highlighted the importance of controlling the water–cement ratio and sand replacement level. Li et al. [12] further revealed the multi-scale relationship between aeolian sand characteristics and concrete strength. Liao et al. [13] found that a high desert sand substitution ratio may increase concrete porosity and widen the interfacial zone, which is detrimental to strength and durability. These studies indicate that desert sand may exhibit a dual effect: a beneficial filler effect at a suitable content and an adverse effect associated with excessive fine particles and weak interfacial bonding.

Coal gangue is another promising alternative aggregate because it is a major solid waste produced during coal mining and washing. Large quantities of coal gangue stockpiles occupy land resources and may cause environmental problems. The use of coal gangue as fine aggregate in cement-based materials can reduce natural aggregate consumption and promote solid-waste utilization. Wang et al. [14] reported that the grading of coal gangue aggregates significantly affects the density, water absorption and strength of concrete. However, compared with natural sand, coal gangue particles usually have higher porosity, higher water absorption and lower particle strength, which may increase the vulnerability of mortar to environmental deterioration. Recent studies on coal gangue concrete and mortar also showed that coal gangue particles can alter pore structure, mechanical behavior and freeze–thaw damage evolution [15,16,17]. Therefore, when coal gangue sand is used in mortar, its influence on both mechanical performance and frost resistance should be systematically evaluated.

Freeze–thaw cycles are among the most severe durability actions for cement-based materials in cold regions. During freezing, water confined in capillary pores and microcracks expands and generates hydraulic pressure, crystallization pressure and local tensile stress within the cementitious matrix. Repeated freezing and thawing gradually initiate microcracks, weaken the ITZ, increase pore connectivity and finally cause surface scaling, mass loss and strength degradation. Valenza and Scherer [18,19] reviewed the phenomenology and mechanisms of salt scaling and frost deterioration, emphasizing that freeze–thaw damage is closely related to pore saturation, ice formation and surface scaling. Scherer [20,21] explained that crystallization pressure generated in pores can cause damage in porous materials. Coussy and Monteiro [22] proposed a poromechanical framework for concrete exposed to freezing temperatures, providing theoretical support for understanding freezing-induced internal stress. These studies demonstrate that freeze–thaw deterioration is a progressive damage process controlled by pore structure, saturation degree, microcrack propagation and interfacial bonding.

Microstructural characterization is essential for explaining the macroscopic degradation of cement-based materials. The ITZ between fine aggregate and cement paste is usually considered to be the weakest region in mortar and concrete. Scrivener et al. [23] pointed out that the ITZ differs from the bulk cement paste in porosity, hydration products and microstructural compactness. Diamond [24] emphasized that SEM provides direct visual evidence for identifying pores, cracks, hydration products and interfacial defects in cement paste and concrete. Under freeze–thaw action, microcracks often initiate around pores and weak ITZ regions, then propagate and connect with neighboring defects. Therefore, SEM observations are necessary for linking aggregate characteristics, pore-structure deterioration and strength degradation [25].

Although many studies have examined recycled aggregate, desert sand, or coal gangue aggregate separately, systematic comparisons among mortars prepared with different fine aggregates remain limited. More importantly, compressive-strength change and mass loss represent different aspects of deterioration: the former describes the measured load-bearing response, whereas the latter primarily records surface scaling and material detachment. Collapsing both responses into a single scalar ranking can conceal contradictory behavior, particularly when continued hydration or aging affects strength. A two-dimensional trajectory based directly on absolute compressive strength and mass loss can preserve the measured quantities, display the direction of change with cycling, and distinguish aggregate-dependent deterioration paths without imposing subjective weights or thresholds. Weighted indices may still be informative as auxiliary comparisons, but their conclusions require explicit sensitivity analysis.

In this study, four fine aggregates—coal gangue sand, river sand, desert sand, and standard sand—were used to prepare cement mortars with identical nominal proportions. Compressive strength and mass loss were measured after 0, 25, and 50 FTCs, and SEM was used for qualitative observation of post-exposure, post-compression microstructures. The principal analytical contribution is a parameter-free freeze–thaw damage-trajectory map in the absolute-strength–mass-loss plane. A coupled index integrating non-negative strength loss and normalized mass loss was retained only as an auxiliary analysis, and its sensitivity to weighting and normalization choices was evaluated. The objectives were: (1) to compare the compressive response and absolute residual capacity of mortars prepared with different fine aggregates; (2) to quantify surface-material loss and its repeatability; (3) to identify distinct damage trajectories without forcing the data into a universal rank; and (4) to relate the macroscopic trajectories to aggregate moisture/absorption properties and qualitative SEM features while explicitly recognizing the limitations of the experimental design.

## 2. Materials and Methods

### 2.1. Raw Materials

Portland composite cement (P.C 42.5R), conforming to GB 175-2023 [26], was used as the binder. The cement was supplied by Xinjiang Qingsong Jianhua Chemical Industry (Group) Co., Ltd., Xinjiang, China, and tap water was used for mixing and curing. The chemical composition and physical properties in Table 1 were taken from the manufacturer’s quality-control report rather than re-measured in the authors’ laboratory. The reported chemical data are presented as oxide-equivalent mass fractions. The analytical method and instrument model used by the manufacturer were not specified in the supplied certificate; this limitation is stated to prevent the data from being misinterpreted as independent laboratory measurements by the authors.

**Figure 1:**
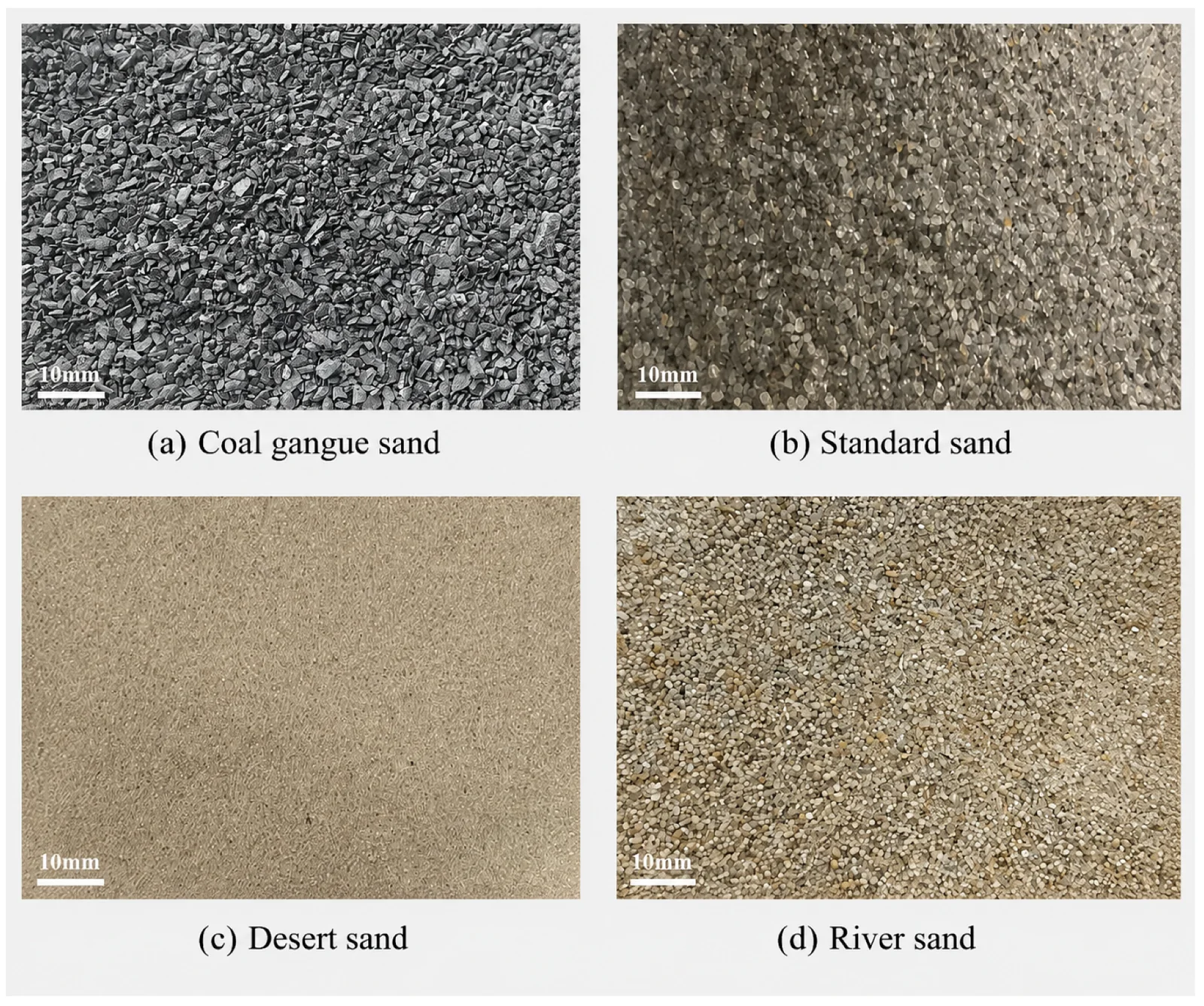
Representative appearances of the four fine aggregates used in this study: (**a**) coal gangue sand, (**b**) standard sand, (**c**) desert sand, and (**d**) river sand.

Four fine aggregates were investigated: coal gangue sand (CGS), river sand (RS), desert sand (DS), and standard sand (StS). RS represented a conventional natural aggregate, StS was used as the reference aggregate, and CGS and DS represented alternative sources with substantially different grading and water-absorption characteristics. Figure 1 shows their representative appearances. The chemical and physical data in Table 2 were supplied in manufacturer/supplier inspection reports. As for the cement report, the supplier certificates did not state the specific analytical instrument model. The oxide values are therefore used descriptively and not as independently verified mineralogical measurements.

The four aggregates differed strongly in grading, packing, and water-related properties. CGS had the largest water absorption (4.50%) and packing void ratio (55.9%), whereas StS had the lowest values (0.35% and 35.8%, respectively). DS had the lowest fineness modulus (1.20), indicating a relatively fine particle-size distribution. The comparatively high CaO content reported for DS (8.59%) may be consistent with the presence of carbonate-bearing constituents such as calcite; however, no X-ray diffraction (XRD) or energy-dispersive spectroscopy (EDS) measurement was available to confirm a particular mineral phase. Accordingly, the CaO result is discussed as a possible compositional influence rather than direct mineralogical proof.

### 2.2. Mixture Proportions and Specimen Preparation

All the mixtures were prepared using the same nominal mass proportions to isolate the effect of aggregate source. The water-to-cement ratio was fixed at 0.45, and the cement:fine aggregate:water ratio was 1:2.87:0.45 (Table 3). These proportions were selected from preliminary trials, were compatible with commonly used engineering mortar proportions, and provided workable mixtures without additional mineral admixtures. Maintaining the same nominal binder, aggregate, and added-water contents also reduced the number of varying factors in the comparative design.

The moisture contents in Table 2 describe the aggregate condition associated with the experiment, and the same nominal added-water content was used for all the mixtures. Because water absorption ranged from 0.35% to 4.50%, the effective water available to the cement paste and the resulting workability and compaction could differ among mixtures even though the nominal w/c ratio was identical. The initial strength differences should therefore not be attributed exclusively to aggregate mineralogy or morphology; differences in water uptake and effective water availability are also plausible contributors [3,27].

Dry cement and aggregate were mixed for 2 min, after which water was gradually added and mixing continued until the fresh mortar was visually homogeneous. The mortar was cast in 70.7×70.7×70.7 mm cubic molds and compacted on a vibrating table. This cube size is specified for construction-mortar compressive testing in JGJ/T 70-2009 and was compatible with the available molds and compression apparatus [28]. Cementitious materials exhibit specimen-size and shape effects because measured compressive strength depends on heterogeneity, boundary restraint, and the probability of critical defects [29]. Therefore, the reported strengths are specific to the 70.7 mm cube geometry and are intended for comparisons within this study; they should not be directly converted to values from other specimen geometries without a validated conversion relationship.

Three cubes were tested for each mixture and FTC condition. The same specimens provided the replicate mass-loss and compressive-strength results; after compressive failure, representative fragments were selected from the central regions of the fractured cubes for SEM observation. No separate unloaded SEM specimens were available. After casting, specimens were covered to limit evaporation, demolded after 24 h, and cured at (20±2) °C and relative humidity above 95%. Freeze–thaw exposure began after 28 d of standard curing.

### 2.3. Freeze–Thaw Cycle Test

**Figure 2:**
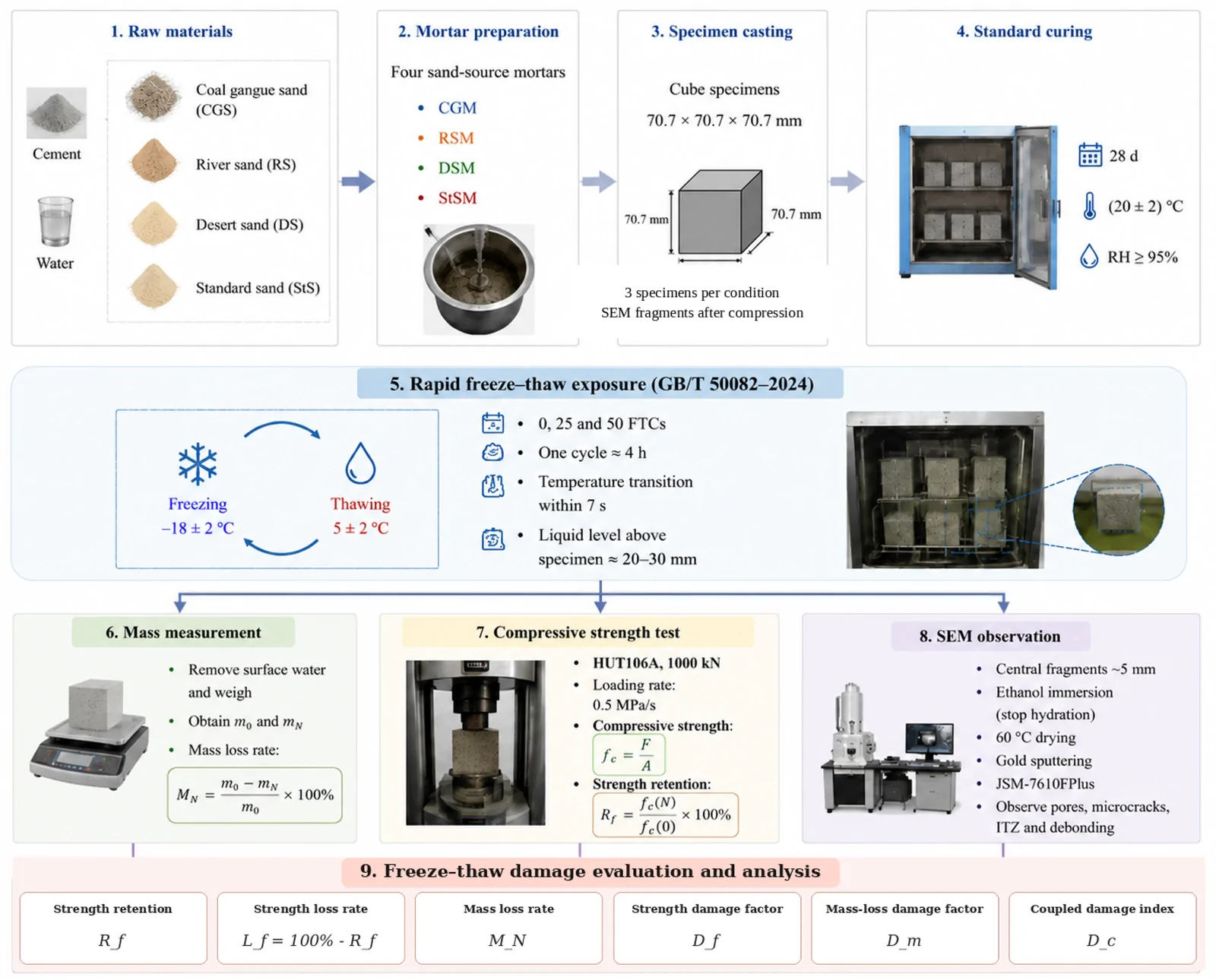
Experimental workflow for material characterization, mortar preparation, specimen casting and curing, freeze–thaw exposure, mass measurement, compressive-strength testing, SEM observation, and freeze–thaw damage evaluation. Arrows indicate the experimental sequence; mixture abbreviations and colors are used consistently throughout the manuscript.

Rapid freeze–thaw testing was conducted in accordance with GB/T 50082-2024 [30]. The specimens were assigned to 0, 25, and 50 FTC conditions. Each cycle lasted approximately 4 h; the central specimen temperature was controlled at (−18±2) °C during freezing and (5±2) °C during thawing, with the freezing/thawing transition completed within 7 s. The liquid level was maintained approximately 20–30 mm above the top of the specimens. At the specified cycle number, surface water was wiped off and loose surface particles were gently removed before weighing and compression testing. Figure 2 summarizes the experimental procedure.

The zero-cycle reference specimens were tested at 28 d, whereas the 25- and 50-cycle specimens were necessarily older when tested. No age-matched non-frozen control specimens were available. Consequently, the measured changes after cycling combine the effects of freeze–thaw exposure with continued aging and hydration. This limitation is especially important when interpreting the apparent strength increase of CGM.

### 2.4. Compressive-Strength Test

Compressive strength was measured using an HUT106A electro-hydraulic servo universal testing machine (Shenzhen Wance Testing Machine Co., Ltd., Shenzhen, China) with a 1000 kN capacity according to GB/T 50081-2019 [31]. The loading rate was 0.5 MPa/s. Three replicate cubes were tested for each mixture and FTC condition, and the results are reported as mean ± sample standard deviation (SD). Compressive strength was calculated as

(1) $$f_{c}=\frac{F}{A},$$

where *F* is the maximum failure load (N) and A=4998.49 mm^2^ is the nominal loaded area. The strength retention and loss rates were calculated as

(2) $$R_{f}\left(N\right)=\frac{f_{c}\left(N\right)}{f_{c}\left(0\right)}\times 100\%,\;L_{f}\left(N\right)=\left[1-\frac{f_{c}\left(N\right)}{f_{c}\left(0\right)}\right]\times 100\%.$$

Only compressive strength was measured. Direct shear testing was not performed, so the influence of FTCs on mortar shear strength cannot be quantified from the present data. Any implication for shear behavior is therefore outside the experimentally supported scope of this work.

### 2.5. Mass-Loss Rate Measurement

The same three replicate specimens used for compressive testing were weighed before and after the specified FTCs. After removal from the chamber, surface water was wiped off and visibly detached particles were removed consistently. The mass-loss rate was calculated as

(3) $$M_{N}=\frac{m_{0}-m_{N}}{m_{0}}\times 100\%,$$

where $m_{0}$ is the initial specimen mass and $m_{N}$ is the mass after *N* cycles. The mass-loss results are reported as mean ± SD. This index predominantly reflects surface scaling, edge damage, and detachment and should not be assumed to represent internal cracking in a one-to-one manner [18,19,32,33].

### 2.6. SEM Observation

SEM observations were made for all four mortars after 25 and 50 FTCs. Representative fragments approximately 5 mm in size were collected from the central region of specimens after compressive fracture. The fragments were immersed in anhydrous ethanol to arrest hydration, dried at 60 °C, sputter-coated with gold, and examined using a JSM-7610FPlus Schottky field-emission scanning electron microscope (JEOL Ltd., Tokyo, Japan). The observations focused on pores, matrix texture, cracks, and aggregate–paste interfacial transition zones (ITZs).

Because all the SEM fragments were obtained after compression, some cracks and interfacial separations may have formed or widened during mechanical loading. No untested zero-cycle SEM reference set or quantitative image analysis was available. The images are therefore interpreted qualitatively as features consistent with the combined exposure and failure history rather than as definitive proof that every observed defect was caused solely by freeze–thaw action.

### 2.7. Freeze–Thaw Damage-Trajectory Analysis

To preserve the directly measured responses and avoid imposing a subjective scalar ranking, each mixture *i* was represented at cycle level *N* by the two-dimensional state vector

(4) $$T_{i}\left(N\right)=\left[f_{c,i}\left(N\right),\;M_{i}\left(N\right)\right],$$

where the horizontal coordinate is absolute compressive strength and the vertical coordinate is mass loss. The mean states at 0, 25, and 50 FTCs were connected in chronological order to form a damage trajectory. Horizontal and vertical error bars denote the corresponding sample SDs. Marker area was scaled to the measured water absorption of the associated aggregate to provide material context; absorption was not used to calculate the trajectory. A movement toward lower strength and/or higher mass loss indicates a less favorable measured response, but no arbitrary thresholds, weights, or universal ranking were imposed. The trajectory map is therefore a descriptive parameter-free integration of the two primary quantitative datasets.

### 2.8. Auxiliary Coupled Damage Index

For comparison with conventional scalarized evaluations, strength change and surface mass loss were also integrated using an auxiliary coupled index. To avoid treating the apparent strength gain of CGM as “negative damage”, the non-negative strength-based factor was defined as

(5) $$D_{f}^{+}\left(N\right)=\max\left[0,1-\frac{f_{c}\left(N\right)}{f_{c}\left(0\right)}\right].$$

The normalized mass-loss factor was

(6) $$D_{m}\left(N\right)=\frac{M_{N}}{M_{\lim}},$$

where $M_{\lim}=5\%$ was used as a reference normalization value. The auxiliary index was

(7) $$D_{c}\left(N\right)=\alpha D_{f}^{+}\left(N\right)+\beta D_{m}\left(N\right),\;\alpha +\beta =1.$$

A baseline combination of α=0.7 and β=0.3 was retained to place greater weight on load-bearing capacity. These choices are not universal material constants. Sensitivity analyses were therefore conducted over α=0.50–0.90 (β=1−α) and $M_{\lim}=3$–7%. The index was used only to test how scalarization changes interpretation; it was not used as the principal result or as a prediction model. The previously included two-point power-law model was removed because two positive cycle observations cannot validate a predictive relationship.

### 2.9. Statistical Analysis

All the quantitative values are presented as mean ± sample SD for n=3. The error bars in the figures represent SD. Two-way analysis of variance (ANOVA) was used to examine the effects of aggregate type, FTC level, and their interaction on compressive strength. Mass loss was analyzed at 25 and 50 FTCs because the zero-cycle value was defined as zero for all the groups. Tukey’s honestly significant difference procedure was used for pairwise comparisons at a significance level of p<0.05. Owing to the small replicate number, statistical inference is treated as supportive rather than definitive. The SD of $D_{c}\left(50\right)$ was estimated from all combinations of replicate baseline strength, 50-cycle strength, and 50-cycle mass-loss values.

## 3. Results and Discussion

### 3.1. Compressive Strength and Repeatability

**Figure 3:**
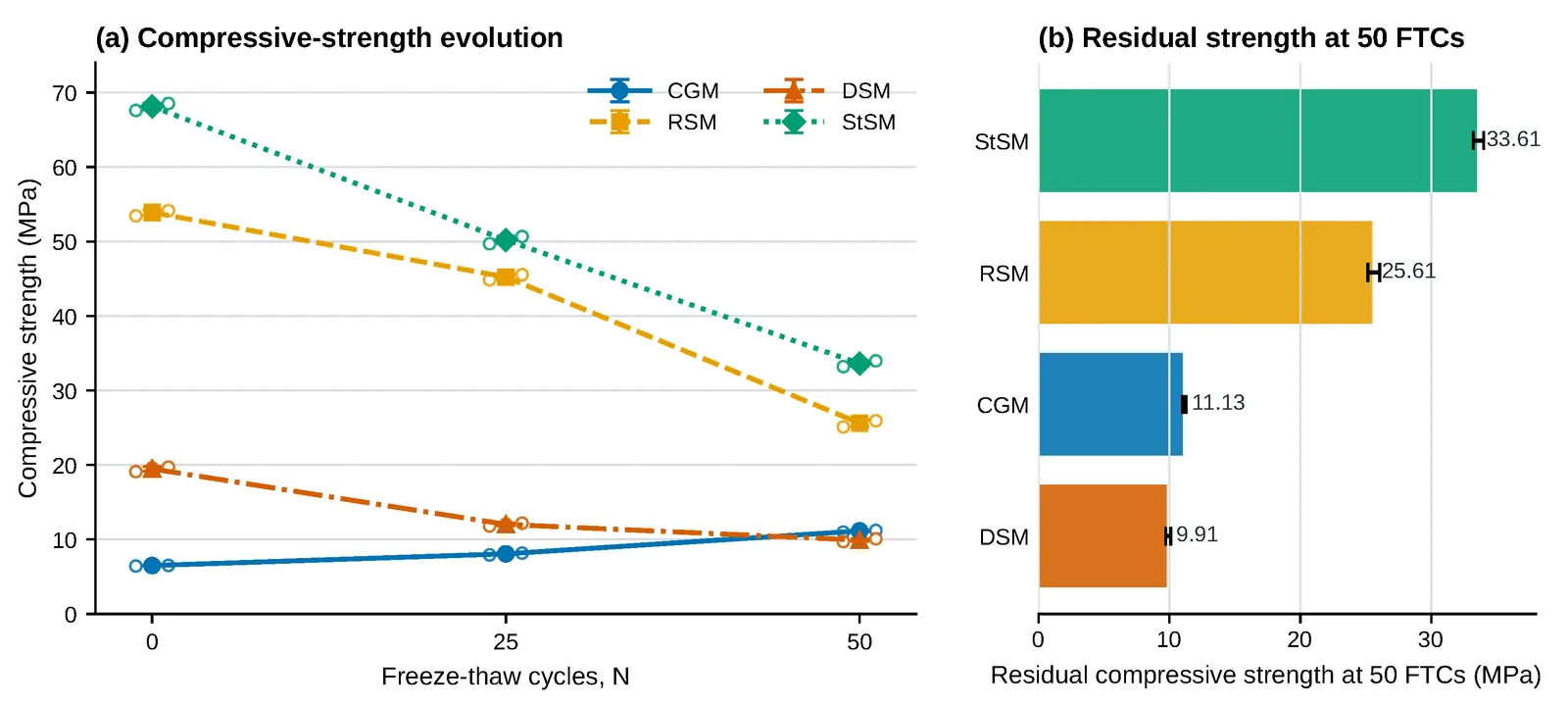
Compressive-strength response across freeze–thaw exposure: (**a**) absolute compressive strength and (**b**) strength-retention response.

Table 4 and Figure 3 present the compressive-strength results. The replicate variability was low, with SD values of 0.07–0.49 MPa. Aggregate type, FTC level, and their interaction were statistically significant (p<0.001), indicating that the strength response to cycling depended strongly on sand source. The Tukey comparisons showed significant differences among the four mixture means within each FTC condition (p<0.05).

At 28 d and before cycling, StSM had the highest strength, followed by RSM, DSM, and CGM. The result is consistent with the combined influence of packing, surface condition, and effective water availability. StS had the lowest water absorption and packing void ratio, whereas CGS had the highest values; thus, a larger fraction of the added water could be taken up by CGS and its pores during mixing. The low initial CGM strength should therefore be interpreted as the outcome of several coupled factors rather than as evidence of a single mechanism.

RSM, DSM, and StSM showed progressive strength reductions. After 50 FTCs, their strength retention ratios were 47.52%, 50.87%, and 49.33%, respectively. Similar percentage losses did not imply similar service capacity: StSM retained 33.61 MPa, RSM retained 25.61 MPa, and DSM retained only 9.91 MPa. Absolute residual strength is consequently essential when mixtures begin from substantially different baselines.

CGM increased from 6.49 to 11.13 MPa. This apparent increase should not be described as improved freeze–thaw resistance. Continued hydration, redistribution of water stored in the highly absorptive aggregate, and age-related strengthening may have competed with freeze–thaw damage [27]. Because the study did not include age-matched non-frozen controls, the separate contributions cannot be quantified. The mass-loss and SEM observations below indicate that physical deterioration occurred despite the higher measured compressive strength.

### 3.2. Mass Loss and Surface Deterioration

**Figure 4:**
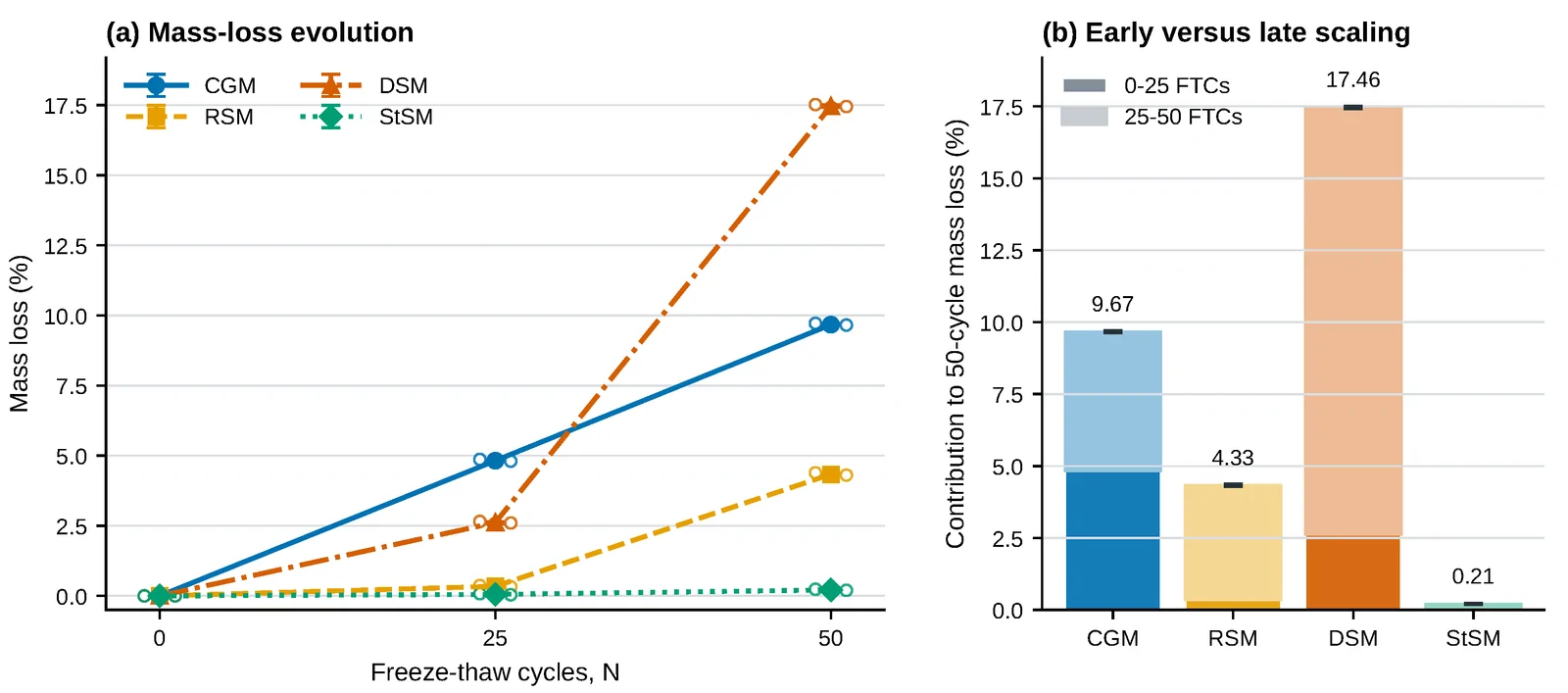
Surface-material loss rate after different freeze–thaw exposures (mean ± SD, n=3).

Table 5 and Figure 4 show the mass-loss results. The SDs were 0.02–0.05 percentage points. For the 25- and 50-cycle data, aggregate type, FTC level, and their interaction were significant (p<0.001); all the mixture means differed within each cycle level according to the Tukey comparisons (p<0.05).

StSM retained excellent surface integrity, with only 0.21% mass loss after 50 FTCs. RSM showed little loss at 25 cycles but reached 4.33% at 50 cycles, suggesting accelerated surface deterioration during the later interval. CGM lost 4.82% by 25 cycles and 9.67% by 50 cycles. Its high aggregate absorption and packing void ratio are consistent with greater local water storage and increased susceptibility to particle detachment, although the experiment did not directly measure saturation gradients. DSM showed the strongest acceleration, increasing from 2.61% to 17.46%. The very fine grading of DS may increase water demand and create heterogeneous paste coverage when a common nominal mix is used; these factors provide a plausible explanation for the pronounced scaling but were not measured independently.

### 3.3. Freeze–Thaw Damage Trajectories

**Figure 5:**
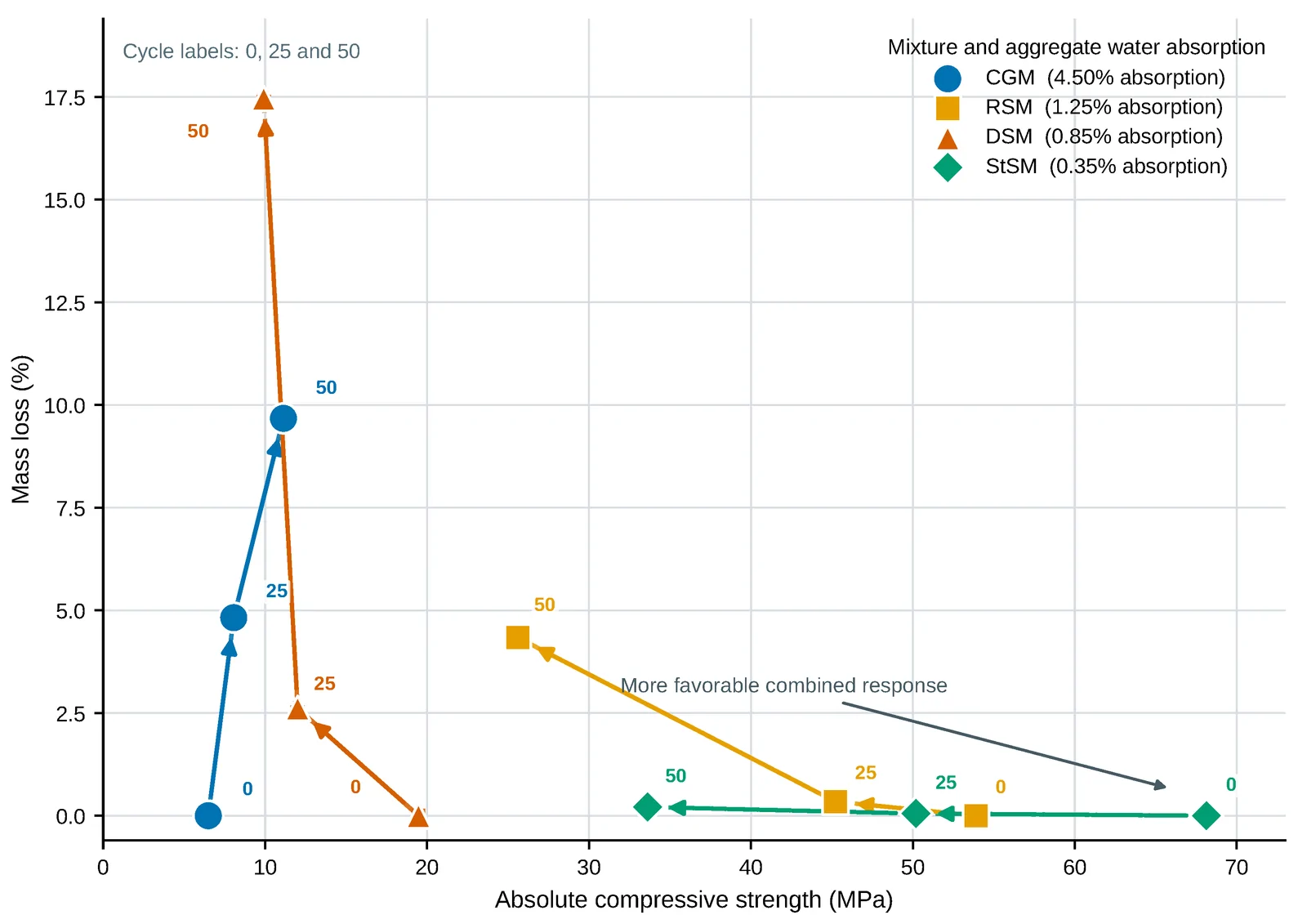
Parameter-free freeze–thaw damage trajectories in the absolute-compressive-strength–mass-loss plane. Arrows connect the mean states at 0, 25, and 50 FTCs; horizontal and vertical error bars denote sample SD (n=3). Marker area is scaled to aggregate water absorption (CGS 4.50%, RS 1.25%, DS 0.85%, and StS 0.35%) for material context only and does not affect the trajectory calculation.

Figure 5 integrates the two primary quantitative responses without converting them into a weighted score. The x-axis retains absolute compressive strength, the y-axis retains mass loss, and arrows show the chronological movement from 0 to 25 to 50 FTCs. This representation makes the direction and mode of change visible while preserving the different physical meanings and units of the measured variables.

StSM moved mainly leftward, from (68.13,0.00) to (33.61,0.21) in the strength–mass-loss plane. Its pronounced reduction in measured compressive strength was accompanied by negligible surface-material loss. Thus, its externally visible scaling resistance was substantially better than its strength retention, demonstrating why mass loss alone would overstate its overall mechanical stability.

RSM followed a leftward trajectory with a moderate upward component. The first 25 FTCs produced a 16.08% strength reduction but only 0.34% mass loss, whereas the second interval ended at 25.61 MPa and 4.33% mass loss. This path indicates that the measured strength response preceded the larger increase in surface scaling. DSM moved from 19.47 MPa and zero loss to 12.01 MPa and 2.61% loss and then sharply upward to 9.91 MPa and 17.46% loss. The late-stage vertical displacement makes its accelerated surface deterioration immediately visible and distinguishes it from the nearly horizontal StSM path.

CGM exhibited the most atypical trajectory: both measured compressive strength and mass loss increased, moving from (6.49,0.00) to (11.13,9.67). This up-and-right path must not be interpreted as simultaneous durability improvement and deterioration. The strength result is confounded by continued aging, hydration, and water redistribution because age-matched non-frozen controls were not included, while the mass-loss coordinate directly demonstrates progressive material detachment. The high CGS absorption, represented by the larger markers, provides relevant context but does not establish causality. Collectively, the four paths show that aggregate source changed not merely the magnitude but also the mode of the measured freeze–thaw response. The trajectory map is therefore used as the principal integrative result of this study.

### 3.4. Auxiliary Coupled Damage Index and Sensitivity

**Figure 6:**
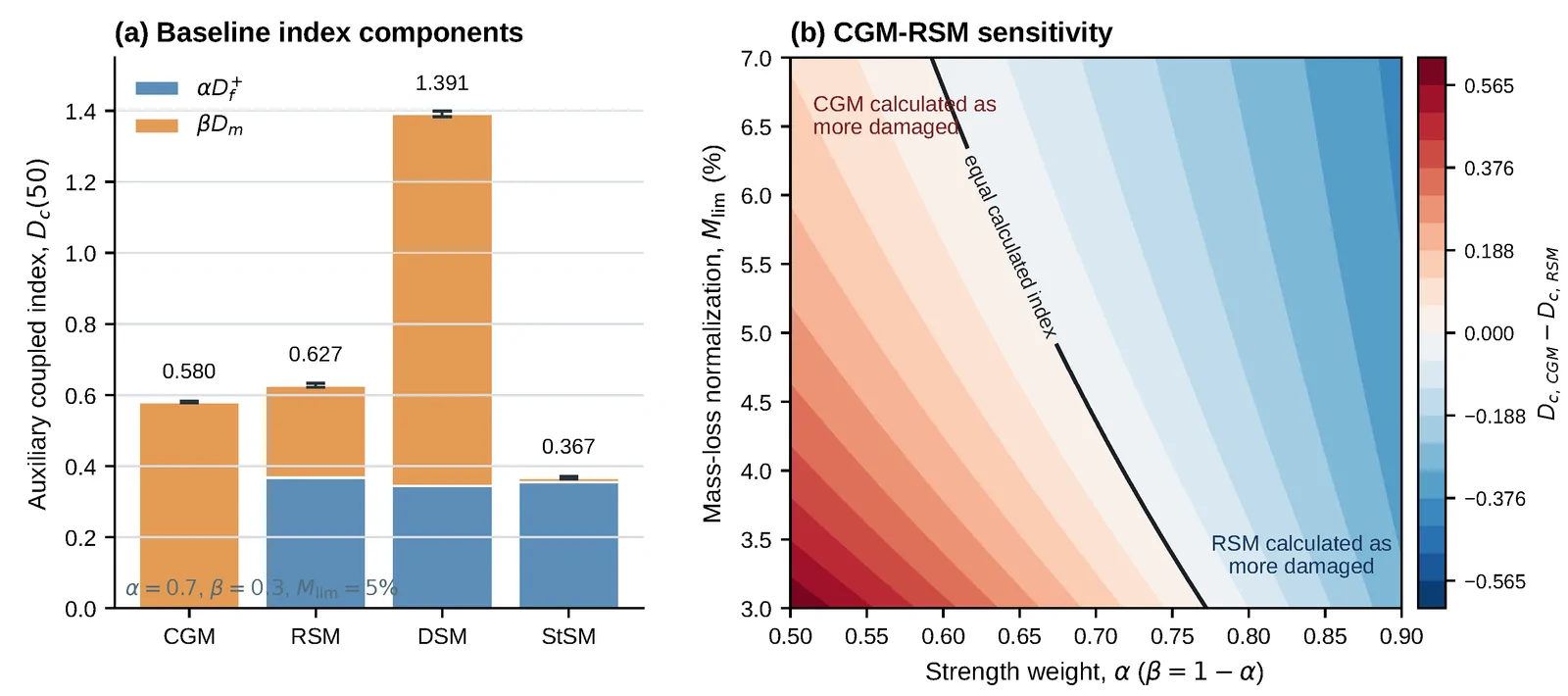
Auxiliary coupled-index analysis at 50 FTCs: (**a**) contributions of the strength-based and mass-loss-based terms under the baseline parameters; (**b**) sensitivity of the calculated CGM–RSM comparison to the strength weight α and mass-loss normalization $M_{\lim}$. The zero contour marks equal calculated indices, and crossing it reverses the scalarized ordering.

The coupled index was retained only to examine the consequences of converting the two measured axes into one number. The baseline components and totals are summarized in Table 6 and Figure 6a. DSM had the largest baseline value because both its non-negative strength-loss component and normalized mass-loss component were high. StSM had the smallest value because its surface-loss contribution was very small despite an appreciable strength-loss contribution. CGM and RSM had similar totals but fundamentally different component structures: CGM’s value was entirely controlled by mass loss because its apparent strength gain was truncated at zero, whereas RSM contained both strength- and mass-loss contributions.

Figure 6b shows that the comparison between CGM and RSM was parameter-dependent. At $M_{\lim}=5\%$, the calculated CGM index was 0.967 at α=0.50, 0.580 at α=0.70, and 0.193 at α=0.90, whereas the corresponding RSM values were 0.696, 0.627, and 0.559. Thus, increasing the strength weight moved CGM from a larger calculated value than RSM to a smaller value because CGM’s strength term was set to zero. Variation in $M_{\lim}$ likewise shifted the equality boundary. This sensitivity confirms that the weighted index is not an intrinsic material property and cannot support a universal durability rank. It is retained as a transparent auxiliary check, while the direct strength and mass-loss data and their parameter-free trajectories remain the primary evidence.

### 3.5. SEM Observations and Microstructural Interpretation

**Figure 7:**
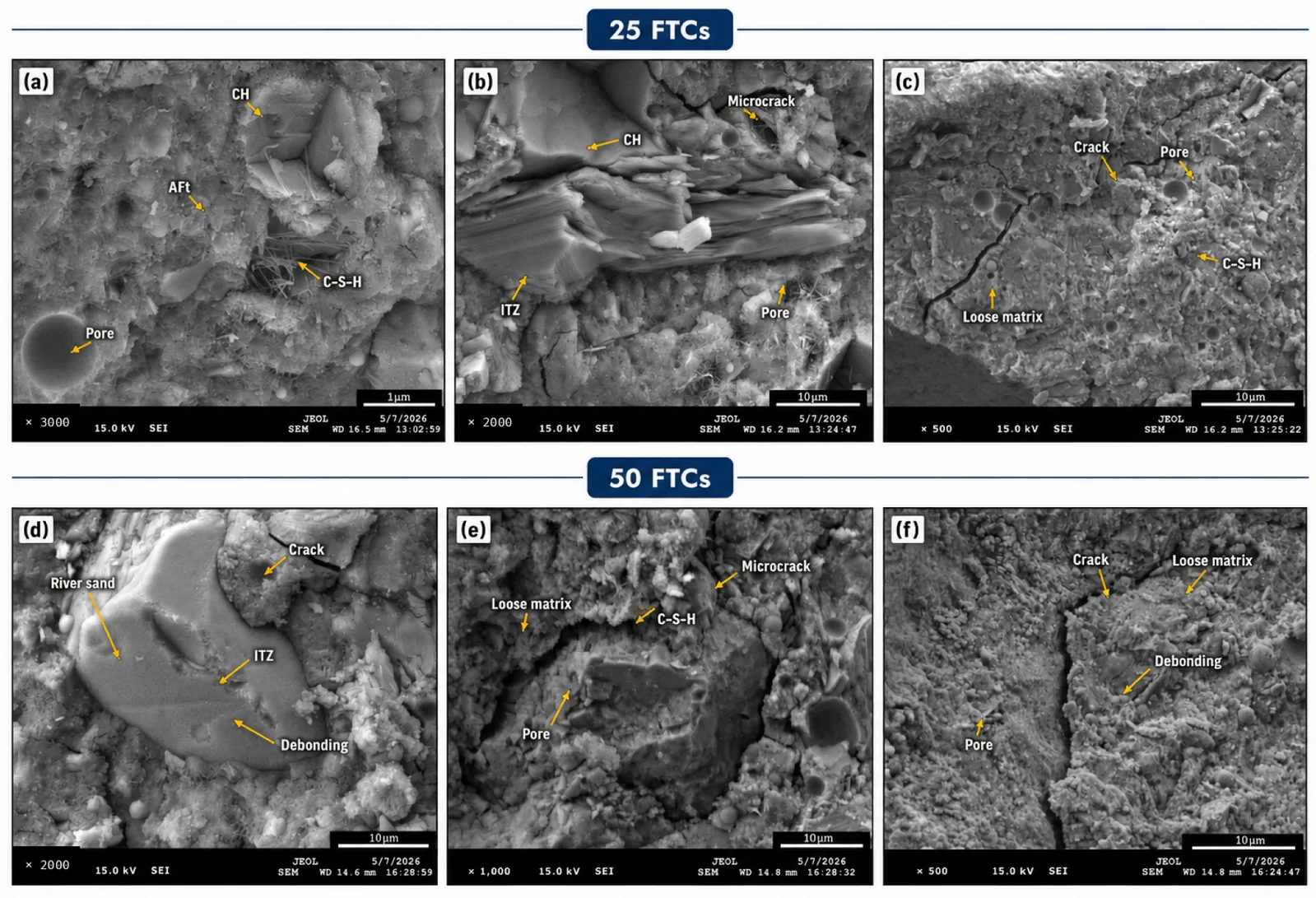
Representative SEM images of RSM: (**a**–**c**) after 25 FTCs and (**d**–**f**) after 50 FTCs. Fragments were obtained after compression testing; labels identify observed morphology and do not uniquely attribute feature origin to freeze–thaw exposure.

**Figure 8:**
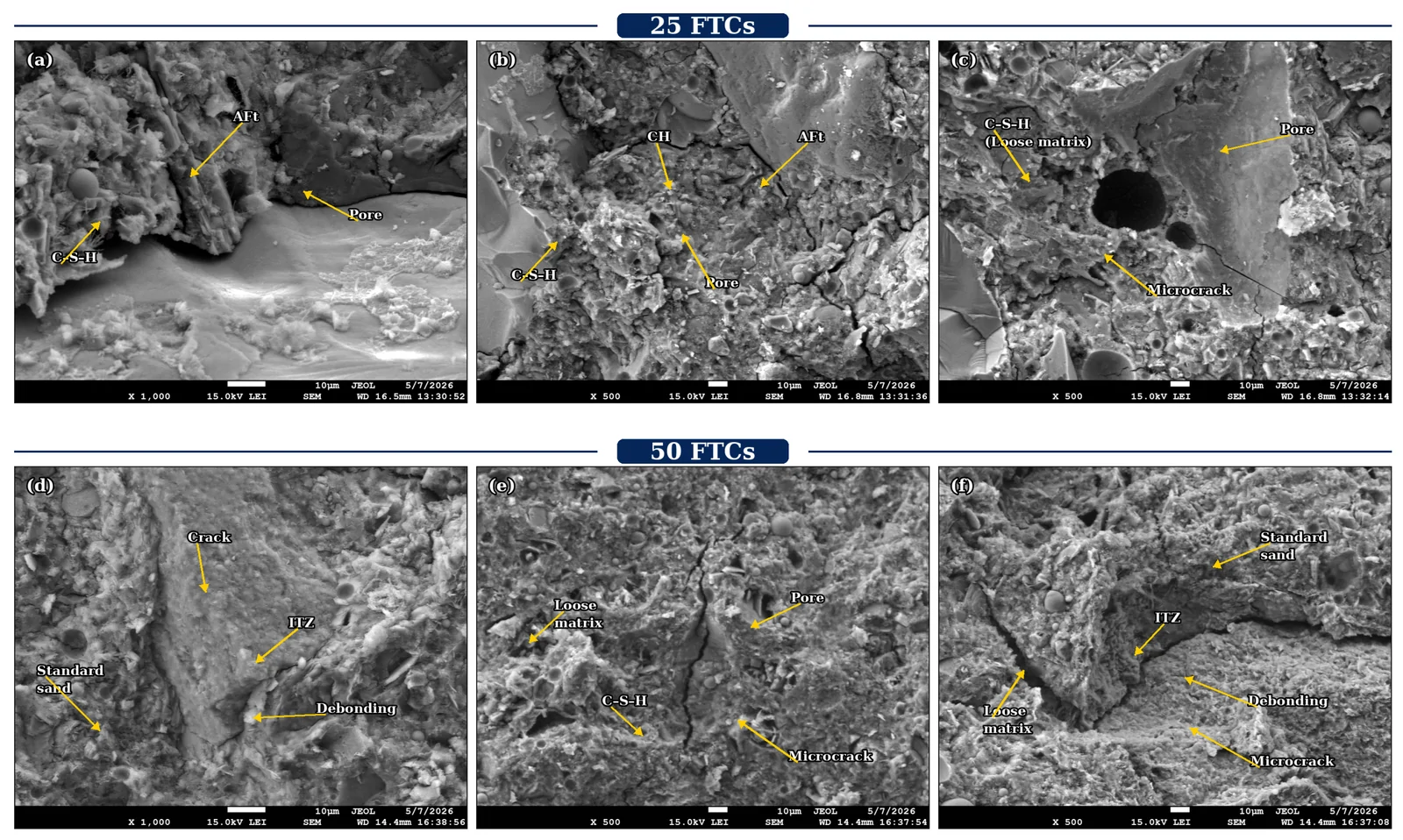
Representative SEM images of StSM: (**a**–**c**) after 25 FTCs and (**d**–**f**) after 50 FTCs. Fragments were collected after compression testing; labels describe observed morphology only.

**Figure 9:**
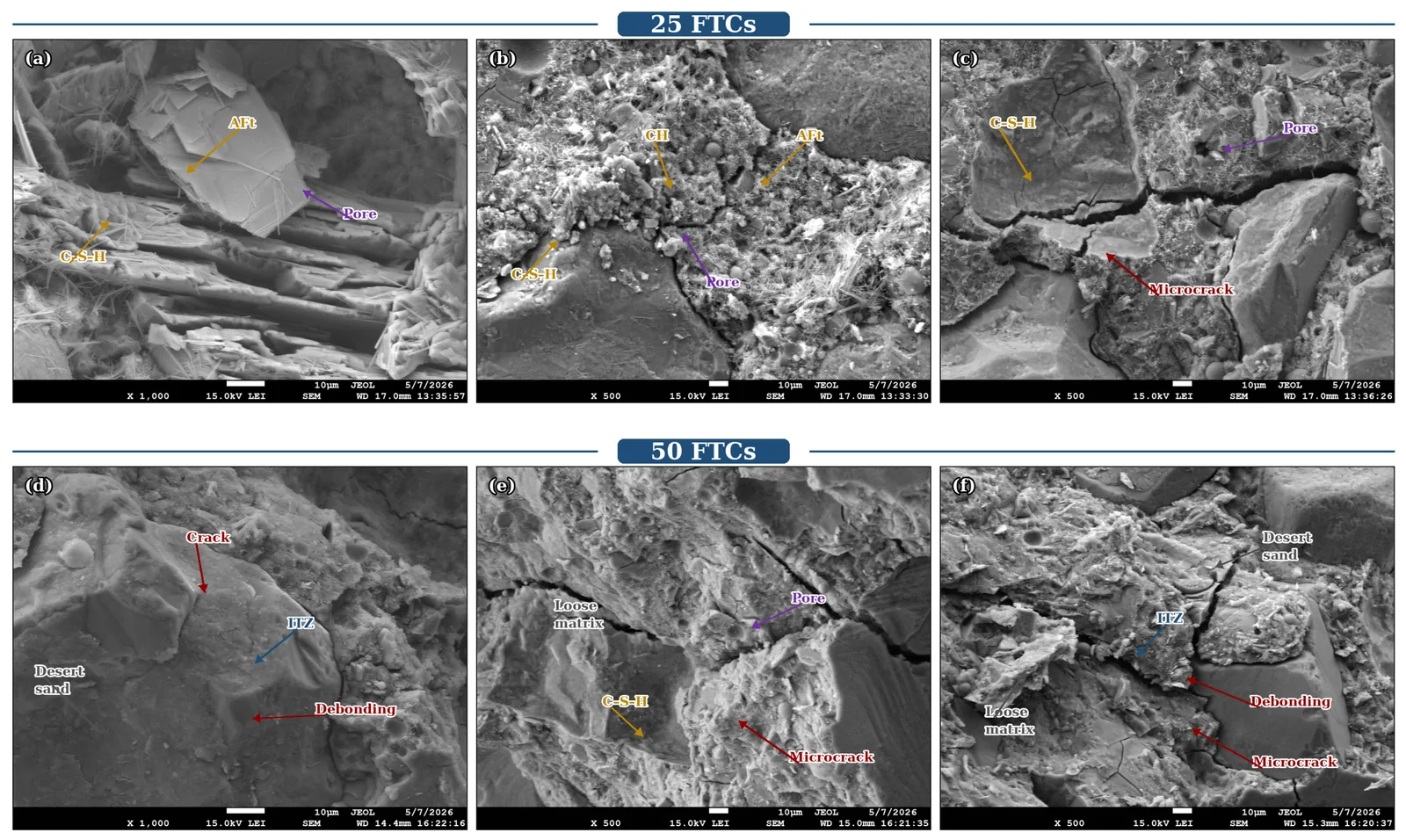
Representative SEM images of DSM: (**a**–**c**) after 25 FTCs and (**d**–**f**) after 50 FTCs. Fragments were collected after compression testing; labels describe observed morphology only and do not uniquely identify mineral phases.

**Figure 10:**
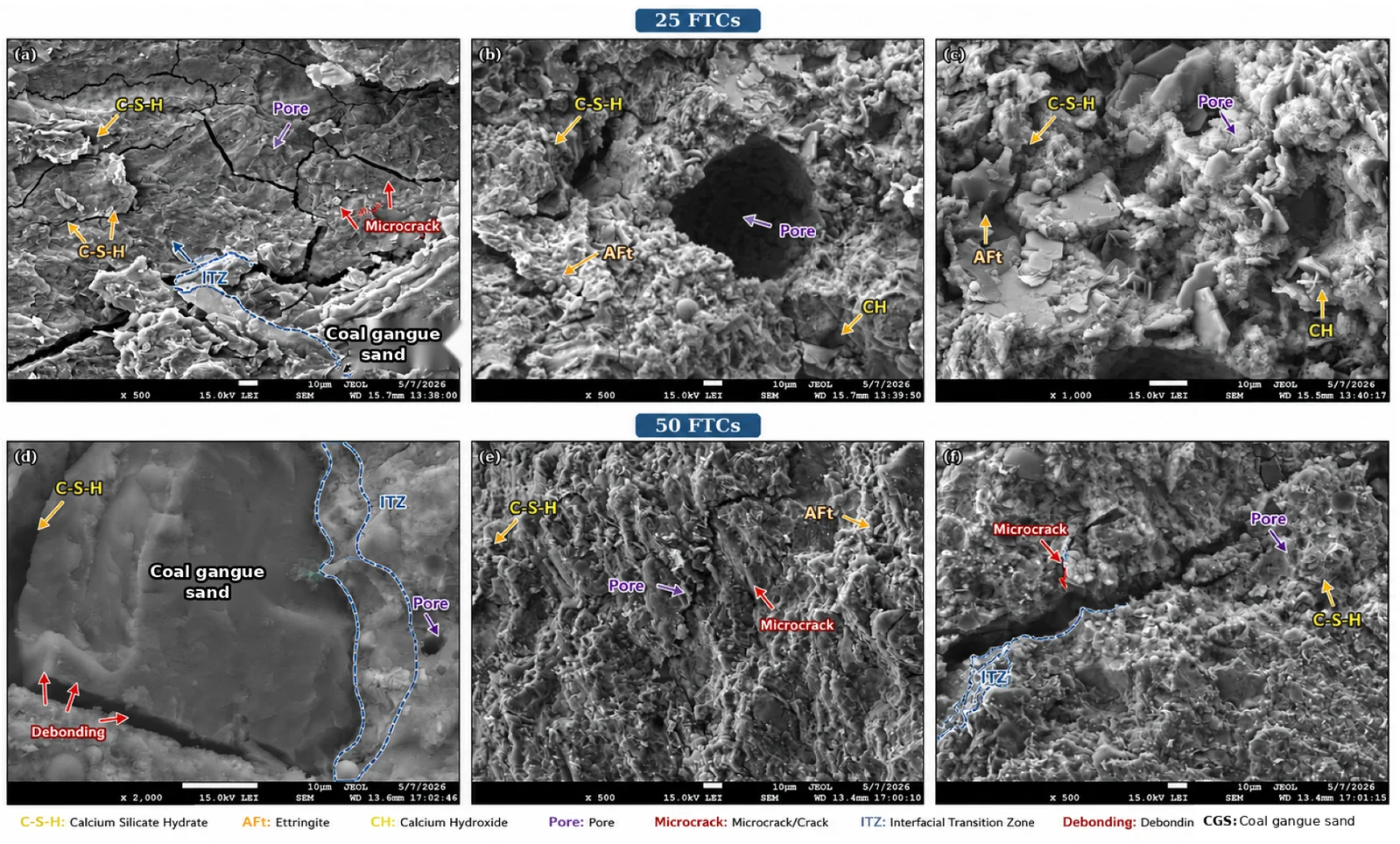
Representative SEM images of CGM: (**a**–**c**) after 25 FTCs and (**d**–**f**) after 50 FTCs. Samples were post-compression fragments; the legend uses the full term “coal gangue sand” rather than an unused abbreviation.

Figure 7, Figure 8, Figure 9 and Figure 10 show representative SEM images. In every figure, panels (a)–(c) correspond to 25 FTCs and panels (d)–(f) correspond to 50 FTCs. Because the samples were collected after compression, the discussion uses cautious terms such as “consistent with” and “may indicate”; it does not assign every crack exclusively to freeze–thaw action.

#### 3.5.1. River Sand Mortar

After 25 FTCs, RSM contained hydration products together with pores and localized microcracks (Figure 7a–c). After 50 FTCs, wider cracks, loose matrix regions, and aggregate–paste separation were visible in several fields (Figure 7d–f). The progression is consistent with reduced interfacial continuity and helps to explain the decrease from 53.90 to 25.61 MPa. Nevertheless, the relatively moderate 4.33% mass loss indicates that much of the damage had not translated into the degree of surface detachment observed for DSM.

#### 3.5.2. Standard Sand Mortar

StSM showed pores and localized cracks at 25 FTCs, followed by more visible cracking and ITZ separation at 50 FTCs (Figure 8). These observations are consistent with the measured strength reduction. At the same time, its mass loss remained only 0.21%, indicating that crack development did not cause extensive external scaling. This behavior illustrates the distinction between internal load-bearing degradation and loss of surface material.

#### 3.5.3. Desert Sand Mortar

DSM displayed plate-like particles, pores, and crack-like discontinuities at 25 FTCs, with a looser matrix and more extensive apparent interfacial separation at 50 FTCs (Figure 9). Some plate-like particles were approximately 10–50 μm in lateral size. Their morphology may be compatible with clay-bearing or layered mineral constituents, but SEM morphology alone cannot establish mineral identity. Clay- and ceramic-rich layered phases can exhibit orientation-dependent response under compression [34]; this possibility may contribute to local stress concentration, but confirmation would require XRD/EDS and controlled mechanical characterization.

The supplier-reported CaO content of DS was 8.59%, considerably higher than that of the other sands. This may indicate carbonate-bearing phases, including possible calcite, which could influence nucleation and cement hydration. Because no direct phase analysis was conducted, the carbonate interpretation remains a hypothesis. The combined low residual strength and 17.46% mass loss nevertheless show that DSM was the least favorable mixture under the present nominal proportioning and exposure regime.

#### 3.5.4. Coal Gangue Sand Mortar

CGM showed pores, cracks, and discontinuous ITZ regions at both cycle levels (Figure 10). At 50 FTCs, several fields contained interfacial gaps and loose texture despite the higher compressive strength measured at that age. These images are consistent with the mass-loss evidence that the mixture underwent physical deterioration. The high absorption of CGS may have increased local water storage while also supporting continued hydration; the present design cannot separate these competing effects.

### 3.6. Implications and Study Limitations

The damage trajectories show that aggregate source altered the balance between measured load-bearing response and surface-material loss. Aggregate grading, packing void ratio, moisture state, absorption, and ITZ quality provide plausible explanatory context, but the present data do not isolate their individual effects. Saturation, pore-size distribution, mineral phase abundance, and crack density were not directly quantified. Accordingly, the trajectory patterns are measured outcomes, whereas the proposed material mechanisms should be regarded as hypotheses for subsequent testing.

Several design limitations constrain the conclusions. First, no age-matched non-frozen controls were available, so continued hydration and specimen age are confounded with FTC exposure. Second, shear strength was not measured. Third, SEM specimens were obtained after compression, and no untested zero-cycle image set or quantitative image analysis was available. Fourth, the chemical data originated from supplier reports whose analytical instrument details were unavailable. Fifth, the replicate number was three, which supports SD and exploratory statistical comparisons but limits broad inference. Finally, the auxiliary coupled index is sensitive to subjective parameter choices and is therefore secondary to the direct damage-trajectory analysis. Future work should incorporate age-matched controls, direct shear and dynamic-modulus testing, independently characterized aggregate mineralogy, pre-loading SEM or micro-CT observations, more FTC levels, and larger replicate sets.

## 4. Conclusions

This study compared cement mortars prepared with coal gangue sand, river sand, desert sand, and standard sand by combining replicate compressive-strength measurements, mass-loss measurements, and qualitative SEM observations after 0, 25, and 50 FTCs. The principal analytical result is the parameter-free damage-trajectory map, which retains absolute compressive strength and surface-material loss as separate measured axes rather than forcing them into a single rank. The four mortars followed distinctly different paths. StSM moved predominantly toward lower strength while remaining close to zero mass loss, retaining 33.61±0.39 MPa with only 0.21±0.02% loss after 50 FTCs. DSM moved toward both low residual strength and severe surface deterioration, ending at 9.91±0.19 MPa and 17.46±0.05% loss. RSM showed a substantial strength reduction followed by a larger late-stage increase in scaling, whereas CGM followed an atypical up-and-right trajectory in which measured strength increased while mass loss also accumulated.

The CGM trajectory is especially important for interpretation. Its measured strength increased from 6.49±0.07 to 11.13±0.13 MPa, but its mass loss reached 9.67±0.04% and the post-compression SEM images contained pores, cracks, loose regions, and interfacial discontinuities. Because the 25- and 50-cycle specimens were older than the 28 d reference specimens and no age-matched non-frozen controls were available, the apparent strength increase cannot be separated from continued hydration, aging, or water redistribution. Similarly, the SEM observations provide qualitative context but do not prove that every defect was generated by freezing and thawing because the fragments were collected after compressive failure.

The weighted coupled index was therefore demoted to an auxiliary sensitivity analysis. Its component plot illustrated the different origins of similar scalar values, and the CGM–RSM sensitivity map showed that their calculated ordering reversed as the strength weight and mass-loss normalization changed. The index is not an intrinsic durability parameter and should not replace direct reporting of residual strength and surface loss. Within the present experimental limits, the most defensible practical conclusion is that alternative fine aggregates for cold-region mortar should be screened using joint transparent consideration of absolute residual capacity, mass loss, aggregate moisture and absorption, and carefully qualified microstructural evidence. Future work should add age-matched controls, nondestructive dynamic-modulus or ultrasonic monitoring, unloaded zero-cycle microstructural references, quantitative mineralogical and pore-structure measurements, more cycle levels, and larger replicate sets.

## Figures and Tables

**Table 1:** Manufacturer-reported chemical composition and physical properties of the cement.

Chemical Composition (Mass%)		Physical Properties	
Component	Value	Property	Value
SiO_2_	21.86	Specific surface area (m^2^/kg)	342.0
Al_2_O_3_	5.42	Standard consistency (%)	27.8
Fe_2_O_3_	3.71	Initial setting time (min)	146
CaO	61.35	Final setting time (min)	275
MgO	3.68	3 d compressive strength (MPa)	24.6
SO_3_	2.64	28 d compressive strength (MPa)	46.8

**Table 2:** Manufacturer/supplier-reported chemical composition and physical properties of the fine aggregates.

Item	CGS	RS	DS	StS
Chemical composition (mass%, oxide-equivalent basis)				
SiO_2_	58.13	91.05	72.76	98.64
Al_2_O_3_	23.26	3.38	7.41	0.35
Fe_2_O_3_	5.42	0.54	2.29	0.10
FeO	1.21	0.51	0.83	0.06
CaO	3.11	1.03	8.59	0.08
MgO	2.83	0.19	2.04	0.04
K_2_O	2.61	1.94	1.95	0.12
Na_2_O	1.12	0.25	2.41	0.05
Loss on ignition	4.91	1.06	1.62	0.28
Physical properties				
Apparent density (kg/m^3^)	2520	2610	2630	2670
Bulk density (kg/m^3^)	1111.42	1390.16	1551.62	1714.68
Packing void ratio ^*a*^ (%)	55.9	46.7	41.0	35.8
Clay content (%)	3.8	2.1	1.8	0.2
Moisture content (%)	2.10	1.10	0.45	0.20
Water absorption (%)	4.50	1.25	0.85	0.35
Fineness modulus	2.41	2.60	1.20	2.50
Chloride ion content (%)	0.02	0	0	0
Sulfate content (%)	0.18	0.14	0.10	0.03

^*a*^ The previously reported “porosity” is clarified here as the aggregate packing void ratio, calculated as $[1-\left(\rho_{\mathrm{bulk}}/\rho_{\mathrm{apparent}}\right)]\times 100\%$; it does not represent intraparticle porosity.

**Table 3:** Nominal mixture proportions of the mortar specimens.

Mixture ID	Sand Type	Cement	Fine Aggregate	Water	w/c	Sand/Cement
CGM	CGS	1.00	2.87	0.45	0.45	2.87
RSM	RS	1.00	2.87	0.45	0.45	2.87
DSM	DS	1.00	2.87	0.45	0.45	2.87
StSM	StS	1.00	2.87	0.45	0.45	2.87

**Table 4:** Compressive strength of mortars after different freeze–thaw exposures (mean ± SD, n=3).

Mixture ID	0 FTCs (MPa)	25 FTCs (MPa)	50 FTCs (MPa)
CGM	6.49±0.07	8.06±0.12	11.13±0.13
RSM	53.90±0.39	45.23±0.35	25.61±0.45
DSM	19.47±0.33	12.01±0.19	9.91±0.19
StSM	68.13±0.48	50.19±0.49	33.61±0.39

**Table 5:** Mass-loss rates after different freeze–thaw exposures (mean ± SD, n=3).

Mixture ID	0 FTCs (%)	25 FTCs (%)	50 FTCs (%)
CGM	0.00±0.00	4.82±0.04	9.67±0.04
RSM	0.00±0.00	0.34±0.02	4.33±0.05
DSM	0.00±0.00	2.61±0.04	17.46±0.05
StSM	0.00±0.00	0.05±0.02	0.21±0.02

**Table 6:** Auxiliary damage indicators after 50 FTCs; baseline parameters α=0.7, β=0.3, and $M_{\lim}=5\%$.

Mixture ID	$D_{f}^{+}\left(50\right)$	$D_{m}\left(50\right)$	$D_{c}\left(50\right)$, Mean ± SD
CGM	0.000	1.935	0.580±0.002
RSM	0.525	0.867	0.627±0.006
DSM	0.491	3.493	1.392±0.008
StSM	0.507	0.042	0.367±0.004

## Data Availability

The original contributions presented in this study are included in the article. Further inquiries can be directed to the corresponding author.

## Abbreviations

ANOVA	Analysis of variance
CGM	Coal gangue sand mortar
CGS	Coal gangue sand
DSM	Desert sand mortar
DS	Desert sand
EDS	Energy-dispersive spectroscopy
FTCs	Freeze–thaw cycles
ITZ	Interfacial transition zone
RSM	River sand mortar
RS	River sand
SD	Standard deviation
SEM	Scanning electron microscopy
StSM	Standard sand mortar
StS	Standard sand
XRD	X-ray diffraction
